# From infection niche to therapeutic target: the intracellular lifestyle of *Mycobacterium tuberculosis*

**DOI:** 10.1099/mic.0.001041

**Published:** 2021-04-07

**Authors:** Leah Isobella Rankine-Wilson, Tirosh Shapira, Carine Sao Emani, Yossef Av-Gay

**Affiliations:** ^1^​ Department of Microbiology & Immunology, The University of British Columbia, Vancouver, Canada; ^2^​ Division of Infectious Disease, Department of Medicine, The University of British Columbia, Vancouver, Canada

**Keywords:** tuberculosis, phagocytosis, macrophages, Host-Directed Therapy, TB

## Abstract

*
Mycobacterium tuberculosis* (Mtb) is an obligate human pathogen killing millions of people annually. Treatment for tuberculosis is lengthy and complicated, involving multiple drugs and often resulting in serious side effects and non-compliance. Mtb has developed numerous complex mechanisms enabling it to not only survive but replicate inside professional phagocytes. These mechanisms include, among others, overcoming the phagosome maturation process, inhibiting the acidification of the phagosome and inhibiting apoptosis. Within the past decade, technologies have been developed that enable a more accurate understanding of Mtb physiology within its intracellular niche, paving the way for more clinically relevant drug-development programmes. Here we review the molecular biology of Mtb pathogenesis offering a unique perspective on the use and development of therapies that target Mtb during its intracellular life stage.

## Introduction

Tuberculosis (TB) caused by *Mycobacterium tuberculosis* (Mtb), remains the leading infectious killer worldwide despite the intensive and progressive work on development of therapeutics [1]. Tuberculosis kills an estimated 1.7 million people annually, with a global case fatality rate of 23 % and a poor treatment success rate as low as 55 % [1], leading the World Health Organization (WHO) to implement programmes including the Moscow Declaration to End TB, dedicated for the fight against TB by 2030. To further exacerbate the threat TB places on global health and economy, resistance to first-line drug rifampicin (RIF) is seen in 10 % of cases in high-burden countries and multidrug resistance in Mtb is becoming wide spread across the world [1, 2]. With rifampicin being the most effective anti-TB drug currently on the market, it is clear that more effort is needed in developing novel treatments, investigating the mechanisms of resistance of drugs against Mtb and understanding Mtb virulence.

Mtb disables macrophage killing machinery, sequestering itself away from both the innate and adaptive immune system, and notably from chemotherapeutics classically made to target extracellular bacteria. Additionally, the non-vascularized location of pulmonary TB lesions and granulomas make compound accessibility incredibly challenging [3]. This, together with Mtb’s unique microbial characteristics exemplified by its slow growth rate and lipid-rich outer membrane further stunt drug bioavailability. Furthermore, since Mtb often drives a strong cell-mediated immune response, infection often results clinically in extensive inflammation, which is associated with substantial and life-threatening tissue damage. Therefore, development of therapies that not only reduce bacterial burden but also are host compatible and limit associated tissue damage are at the forefront of current research. Indeed, anti-inflammatory agents such as the non-steroidal anti-inflammatory drugs [4] and signalling modulation agents such as metformin [5] provide a promising avenue for a new line of adjunctive TB therapeutics termed host-directed therapies (HDTs) discussed in this paper. HDTs join nano-medicine [6–8] as promising novel treatment options for Mtb infections. Here we review the current state of development of tuberculosis therapies, focusing on novel compounds that act on or inhibit processes manipulated by Mtb inside the host macrophage.

### Phagosome maturation

Macrophage phagosomes undergo a tightly controlled two-step process termed phagosomal maturation, which enables their progressive acidification and terminal fusion with lysosomes. Phagosome maturation initially occurs via the activation of transmembrane vacuolar ATPase pumps, which acidify the early phagosome via proton influx. Next, phagosomes are trafficked into, and merge with a series of increasingly acidified membrane-bound lysosomal vesicles from the Golgi that contain proteases, lipases and other lytic enzymes. The phagosomal pH starts from neutral (~pH 7) and progresses to pH 5.0 in the terminal phagolysosome [9, 10]. The acidic environment facilitates particle degradation and enhances the pH-dependent activation of proteases and other molecules responsible for antigen presentation, such as the removal of class II invariant chain-associated peptide (CLIP) from the major histocompatibility complex class II (MHCII) [11]. Sequential membrane-tagging of the phagosome with docking proteins allow the scale of acidification to rise as the process matures [12–14].

**Fig. 1.**
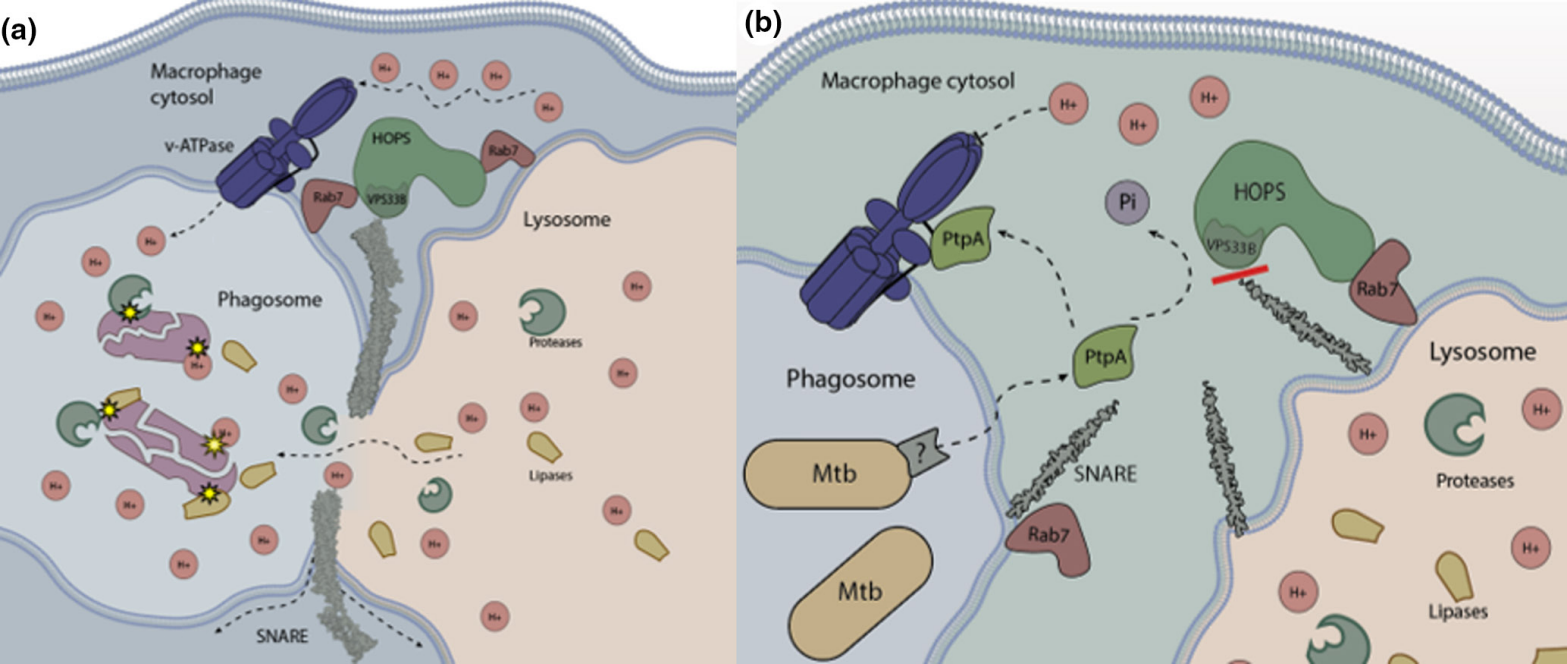
(a) Phagolysosome maturation during phagocytosis. Phagosomes are gradually acidified by the recruitment of the v-ATPase pump, then follows docking of the phagosome to the lysosome via Rab7-HOPS interaction and SNARE tethering facilitating fusion. Phagosome lysosome fusion results in the degradation of the invading microorganism via a number of proteases and lipases. (b) Phagosome maturation arrest mediated by the Mtb secreted phosphatase PtpA. Mtb is able to thwart phagosome maturation using PtpA, which binds to subunit H of the v-ATPase, preventing early phagosome acidification. PtpA also dephosphorylates the HOPS complex subunit VPS33B, inhibiting tight-binding SNARE protein interactions.

Rab GTPases are key directors in membrane trafficking and phagosome maturation. They serve as molecular switches that determine the transitory elements of vesicular intermediates by docking to specific guanine exchange factors (GEFs) [12]. Initially, Rab5 is present on the early phagosomal membrane, allowing docking of the early endosomal antigen 1 (EEA1). EEA1 assists in fusion with the early endosome and as such is used as a typical marker for trans-Golgi associated, non-acidic, early endosomes [15]. Concurrently, the class C core vacuole/endosome tethering (CORVET) complex binds to Rab5 enabling fusion through soluble N‐ethylmaleimide‐sensitive factor attachment protein receptors (SNARE) family protein interactions [12] (Fig. 1a). SNAREs assist in the final stage of membrane fusion in many biological systems and are needed to overcome strong hydrophobic charges associated with the phospholipid membrane in order to tightly bind and merge them [16]. In a successful xenobiotic-clearing pathway, the maturation process proceeds when Rab5 is replaced with Rab7 on the phagosomal membrane and to a lesser extent, with Rab9 [17]. The main role of Rab7 is the tethering of the homotypic fusion and vacuole protein sorting (HOPS) complex, which allows for docking and fusion with the lysosome: the final stage of phagosome maturation (Fig. 1) [Review: 18]). Phagolysosome biogenesis is accelerated by the recruitment of Rab7‐interacting lysosomal protein (RILP) to the phagosomal surface [19], which associates with dynein-dynactin motors causing tubular extensions to facilitate contact and subsequent merging of the phagosome and lysosome [20]. Overall, the phagosome maturation process is a tightly managed process involving the orchestration of cytoskeletal, vascular and nuclear components.

By discussing these mechanisms, we are able to describe the points at which Mtb interferes with phagosome maturation. In brief, Mtb uses an array of lipids, proteins and secreted effectors to exist inside a classically hostile environment [Review: 3]. Contrary to a host-favoured pathogen-clearing cascade and as a result of mycobacterial evasion mechanisms, phagosomes containing Mtb are characterized by the absence of Rab7, v-ATPase, lysosomal associated membrane protein 1 (LAMP1), and EEA1 [21] (Fig. 1b).

### Phagosome maturation arrest

Mtb is able to subvert or neutralize host defenses early in the phagosome maturation pathway using a wide variety of molecules and strategies. Several secreted effector proteins (SapM, PtpA, PknG) and the membrane liposaccharide lipoarabinomannan (LAM), (Table 1), are involved in inhibiting phagosome-lysosome fusion [22–24]. Additionally, the glycolipid-rich component of the mycobacterial cell wall was shown to play an important role in blocking the phagosome maturation process, essentially promoting intracellular survival [13]. Specifically, Mtb’s glycolipids interfere with phagosome-lysosome fusion through blocking of intracellular trafficking, a process partially mediated by the inhibition of host phosphatidylinositol 3-phosphate (PI3P) deposition on the phagosome surface [13, 14]. PI3P is generated in the early stages of the phagocytic cycle by phosphoinositide 3-kinase (PI3K), a well-characterized GEF of Rab5 [25], and is required for successful phagosome maturation [13, 15]. Mtb modulates PI3P signalling in several points in the cascade, from limiting its generation, to active removal from the phagosome surface. PI3P is expressed on the phagosome surface in temporal waves coinciding with one of two stages of early maturation. Mtb reprograms these waves to expresses minimal PI3P uniformly over a temporal scale thus inhibiting the signalling pathway for phagosome maturation [26]. Additionally, Mtb is known to limit PI3P production via host mannose receptor (MR, CD206), which coordinates host SHP-1 to the Mtb-containing phagosome. SHP-1 actively limits PI3P generation and has been associated with promoting Mtb growth, as shown by chemical inhibition of SHP-1 with sodium stibogluconate [27]. Adding to this mechanism, Mtb also prevents PI3P accumulation on the phagosomal membrane by blocking the syntaxin 6-dependent delivery of molecules from the Golgi [14]. This process, mediated by the mycobacterial cell-wall component Mannose-capped LAM (ManLAM), consequently inhibits compartmental trafficking in the cell.

**Table 1.** Mtb macromolecular characteristics associated with infection

Mtb component	Gene name/Rv no.	Description of trait	Reference
**Cell-wall glycolipids**	n/a	Inhibits PI3P deposition on the phagosome surface	[13, 14]
**Ergothioneine (ERG**)	n/a	Low molecular weight thiol that scavenges free radicals	[107]
**ESAT-6**	*esxA* / Rv3875	Prevents the conversion from Rab5 to Rab7, driver of necrosis, secreted protein	[33, 183, 187]
**ESX-secretion system**	n/a	Lysis of host membranes, horizontal gene transfer	[188, 189]
**Gamma-glutamylcysteine (GGC**)	n/a	Low molecular weight thiol that scavenges free radicals	[108]
**GarA**	*garA* / Rv1827	PknG substrate, regulator of glutamate biosynthesis	[72]
**Glutathione**	n/a	Low molecular weight thiol that scavenges free radicals	[108, 190]
**Lipoarabinomannan (LAM**)	n/a	Inhibits early phagosomal markers, scavenges free radicals	[13]
**ManLAM**	n/a	Inhibits PI3P deposition on the phagosome surface	[14]
**Mycothiol (MSH**)	*mshA* and *mshC* /Rv0486 and Rv2130c	Low molecular weight thiol that scavenges free radicals	[105]
**PhoP**	*phoP* / Rv0757	Two-component system response regulator, associated broadly with pathogenesis	[191]
**Protein kinase G (PknG**)	*pknG* / Rv0410c	Kinase associated with metabolic regulation and intracellular survival, inhibits phagosome lysosome fusion	[69, 71, 72]
**Protein kinase A (PtkA**)	*ptkA* / Rv2232	Kinase that increases PtpA activity via phosphorylation	[49, 50]
**Protein tyrosine phosphatase A (PtpA**)	*ptpA* / Rv2234	Inhibits phagosome-lysosome fusion via blocking of v-ATPase and dephosphorylation of HOPS subunit VPS33B	[24, 47, 192]
**Secreted acid phosphatase (SapM**)	*sapM* / Rv3310	Inhibits PI3P deposition on the phagosome surface via PI3P hydrolysis	[22]
**SecA2 secretion system**	*secA2*/Rv1821	Secretes PknG and SapM	[193]
**Superoxide dismutase (A and C**)	*sodA* / Rv3846 *sodC* / Rv0432	Converts O^-^_2_ to H_2_O_2_ and dioxygen to reduce free radicals	[194]
**Thioredoxin TrxB2**	*trxB2*/Rv3913	Disulphide reductase, reduces LMW thiols, associated with PtkA	[119, 120]
**Thioredoxin TrxC**	*trxC* / Rv3914	Disulphide reductase, reduces LMW thiols	[119, 120]

Mtb is also able to utilize a variety of secreted effectors to assist in intracellular survival. For example, the secreted acid phosphatase of Mtb (SapM) (Table 1), hydrolyses PI3P to phosphatidylinositol (PI), thus reducing PI3P accumulation on the phagosome surface and consequently resulting in pleotropic effects that lead to the prevention of phagosome acidification [22]. A recent report defined SapM as an atypical monoester alkaline phosphatase (being most enzymatically active at a pH of 7.5), with a serine-dependent mechanism of catalysis, which showed activity towards important docking intermediates such as phosphatidylinositol 4,5-bisphosphate [PI (4,5)*P*_2_] and PI3P [28]. The construction of a SapM expression strain in *Escherichia coli* has recently been published, allowing further research into its dynamics in both soluble and catalytically active forms [29]. Interestingly, the SapM-mediated reduction of PI3P on phagosome surfaces also aids in the prevention of IL-10-induced autophagy via activation of the signal transducer and activator of transcription 6 (STAT6) pathway. Briefly, IL-10 activate the PI3K pathway, resulting in a mammalian target of rapamycin complex 1 (mTORC1) and Akt/protein kinase B (PKB) activation, inhibiting both the starvation-induced and typical autophagy processes [30]. Additionally, a T_H_2 cytokine response resulting in IL-4 and IL-13 expression has been shown to inhibit anti-Mtb autophagy [31]

As discussed earlier, during the phagocytic maturation pathway, GTPase Rab5 is replaced by Rab7 in a Rab22a-dependent manner [32], where the Rab7 cognate GEF, the HOPS complex, interacts with the phagosome (Fig. 1a). Mtb has been shown to prevent the conversion from Rab5 to Rab7 via expression of the response regulator PhoP and ESX-secretion system-associated early secreted antigenic target ESAT-6 [33]. Although the direct inhibition of Rab7 recruitment has been shown [33], it is important to note that the inhibition of Rab7 binding is thought to be mostly a downstream result of a cascade initiated by the inhibition of Rab5 and EEA1 by the successful prevention of PI3P deposition mediated partly by SapM [13, 15]. SapM is secreted by the SecA2 secretion system [21], which is also responsible for the translocation of the mycobacterial kinase PknG in both Mtb [21] and *M. marinum* [34]. The role of SecA2 in Mtb pathogenesis has been explored. A lysine auxotroph/*secA2* deletion mutant [35] as well as an *fbp*A/*sapM* double knock-out strain [36] have been suggested for a novel live attenuated vaccines. SapM has been shown to be inhibited by ascorbic acid derivatives [l-ascorbic acid (vitamin C) and 2-phospho-l-ascorbic acid], which although structurally similar, have been proposed to act via two different catalytic mechanisms [28]. Importantly, challenge with these substrates does not affect Mtb growth in broth culture, suggesting their target is specific to Mtb’s intracellular phenotype. Although ascorbic acid has previously been shown to inhibit Mtb in broth supplemented with ferrous ions, due to the reactive nature of the associated Fenton reaction [37]; however its effect on intracellular enzymes has not been well elucidated. Fernandez-Soto and collaborators demonstrated l-ascorbic acid and 2-phospho-l-ascorbic inhibited SapM’s catalytic activity by competitive displacement and metal oxidation, respectively. A trial by the Jacobs’ lab demonstrated the promising potential of vitamin C as an adjunctive therapy in mice [38].

Mycobacteria actively recruit and preserve host protein coronin 1, originally identified as P57 [39] and also referred to as tryptophan aspartate-containing coat protein (TACO) [40, 41]. Coronin 1 is a host actin-associated molecule that mediates interactions between the cytoskeleton and plasma membrane in leukocytes. Coronin 1 is released from the phagosomal membrane in order to allow progression of phagosome-lysosome fusion [42] via modulation of calcium flux and associated processes [21].

LAM, a key component of the Mtb cell wall, was shown to inhibit a cascade consisting of cytosolic Ca^2+^ transients: calmodulin, PI3K and EEA1 (43). This pathway is needed for the maturation of phagosomes into phagolysosomes, as EEA1 and Syntaxin 6 aid in the delivery of lytic cargo to the phagosome [15, 43]. Treatment of macrophages with cyclosporin A and FK506, potent inhibitors of calcineurin, resulted in progressive phagosomal maturation [44]. Although whether the prolonged increase of calcium flux is needed to prevent phagosomal maturation throughout infection has been questioned [45], as prolonged calcium fluxes drive apoptosis, a process blocked by Mtb [46], leading to the need for more research on the dynamics of calcium flux in tuberculosis infection.

### PtpA- PtkA

The most notable of Mtb’s secreted proteins is the protein tyrosine phosphatase PtpA, which is essential for the growth of Mtb within human macrophages [47], and for which an array of direct substrates within the host have been described [24, 46, 47]. Mechanistically, PtpA is secreted into the macrophage cytosol where it prevents HOPS complex tethering in infected macrophages [47], and inhibits the macrophage v-ATPase, stalling phagosome acidification [24] (Fig. 1b). More specifically, PtpA dephosphorylates the HOPS subunit VPS33B, inactivating the complex’s ability to anchor SNARE molecules and preventing phagolysosome tethering (Fig. 1b). In parallel, PtpA excludes the host v-ATPase pump on the phagosomal surface through binding to its subunit H, thus blocking subsequent acidification [24]. The binding of PtpA to the subunit H of the v-ATPase is necessary for the subsequent dephosphorylation of VPS33B, suggesting a two-step localization process to arrange PtpA with the HOPS complex [24].

The *ptpA* gene is part of a three-gene operon and is flanked by *ptkA* and a predicted transmembrane protein encoded by *rv2235*. The *ptkA* gene encodes an atypical protein tyrosine kinase [48, 49], one of whose substrates is PtpA. PtkA phosphorylates PtpA on Tyr-128 and Tyr-129, resulting in enhanced PtpA phosphatase activity [49, 50]. Individual knock-out mutants of *ptp*A [47] and *ptk*A [51] both show impaired intracellular survival indicating that these signalling proteins are independently important for Mtb pathogenicity. As outlined earlier, PtpA has been shown to be secreted [47, 52], yet it lacks the necessary signal sequence required for secretion via the Tat or SecA1 systems, leading to the suggestion that its secretion is carried via an alternate secretion pathway [21], however, to date, the secretion system and the exact mechanism behind PtpA secretion still need to be established.

Deletion of the *ptp*A gene showed no effect on Mtb growth *in vitro*, nor on its ability to initiate infection in human cells [24, 47]. However, once phagocytosed, PtpA knock-out mutants show attenuated growth, accompanied by significantly increased events of phagosome-lysosome fusion [47].

A proteomic kinome study analysing host signalling networks affected by Mtb during infection identified the broad effect of Mtb on host signalling, affecting pathways associated with Src, JNK, PKCα, NRI and PKR1, among others [46]. Of particular interest, the glycogen synthase kinase alpha, GSK3α, saw a 60 % decrease in phosphorylation in THP-1 cells infected with wild-type Mtb compared to *∆ptpA* [46], highlighting the key role of host phosphorylation in controlling infection. PtpA is able to dephosphorylate GSK3α at Y279, and phosphorylation of this residue is required for proper enzyme function [46]. PtpA is able to functionally inhibit GSKα’s associated apoptotic signalling in order to increase host cell survival during early Mtb infection [46, 53]. Interestingly, in terms of both macrophage survival and kinome analysis, the PtpA depletion mutant showed high similarity of attenuation and host phosphorylation patterns, respectively, to that of the BCG vaccine strain [54, 55], suggesting exploiting the role of PtpA in mycobacterial virulence could define a novel vaccine strain that more closely exposes the host to Mtb specific epitopes.

**Fig. 2.**
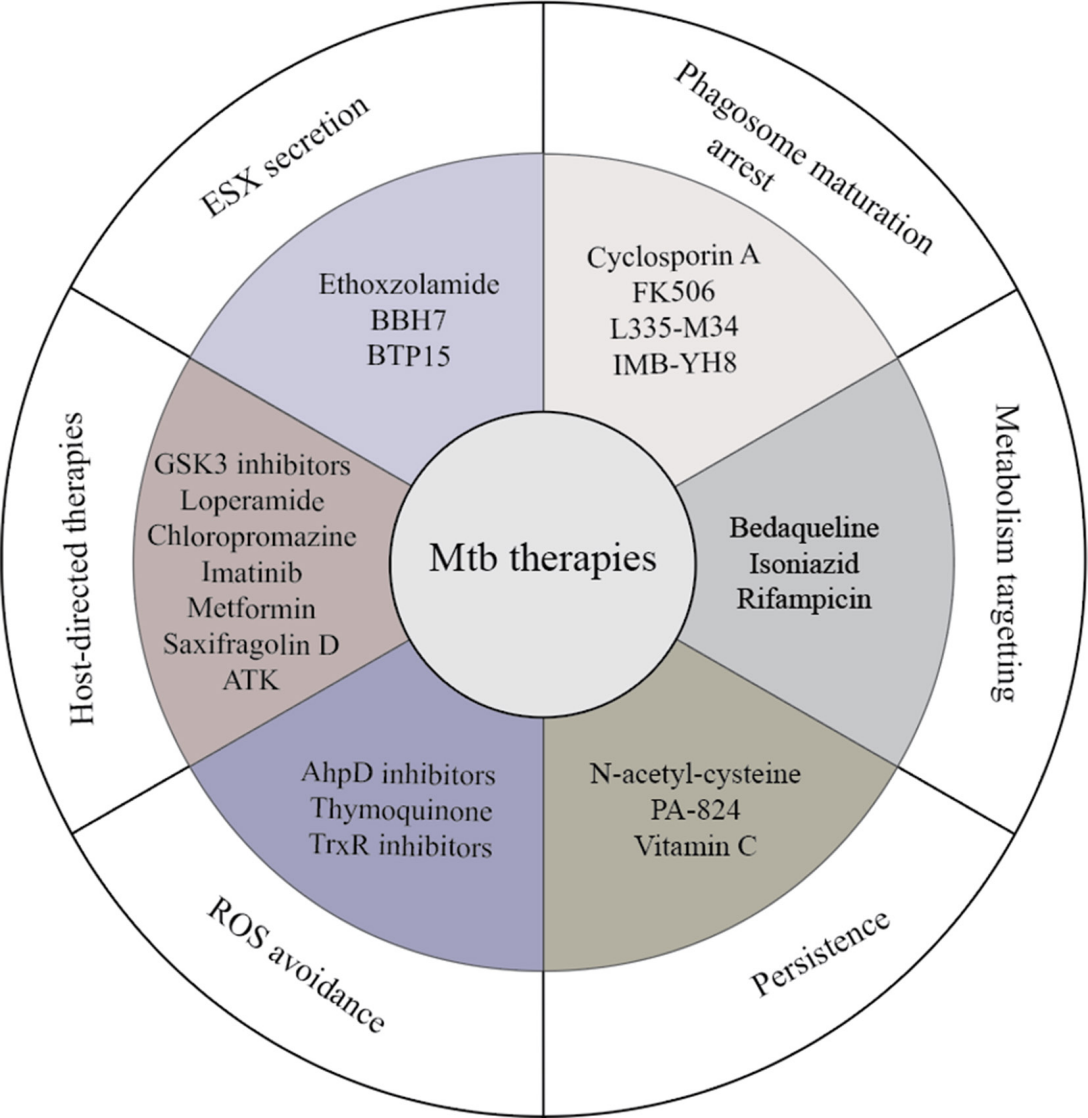
Compounds and targets against intracellular Mtb compounds targeting processes promoting Mtb infection.

Several compounds targeting PtpA have been identified in recent years (Table 2) [56–60]. PtpA inhibitors lack direct antimicrobial activity in broth culture yet are able to facilitate infection clearance. PtpA inhibitors have been able to reduce bacillary load by as much as 77 % after 96 h [61], with low cytotoxicity. Cross reactivity of PtpA inhibitors against human protein tyrosine phosphatases was addressed in several studies, demonstrating the need for compounds with better specificity [60, 62]. A lead compound against PtpA, L335-M34 (Table 2, Fig. 2), has an IC_50_ of 160 nM and shows significant synergy when used in combination with isoniazid (INH), RIF and pyrazinamide in the guinea pig model [60]. A more recent publication demonstrated that activity of PtpA was inhibited by *cis*-2 and *trans*-2 eicosenoic fatty acids, with both compounds showing inhibition of PtpA, with IC50 at 8.20 and 11.26 µM, respectively [63]. Hydrogen bond-mediated binding activity was postulated via *in silico* modelling. Although biologically significant (over log) inhibition was not seen at concentrations up to 30 µM, the data are consistent with other PtpA inhibitors [61]. Evidently, the need for inhibitors acting in the sub-micromolar range is rising, calling for more structure-activity relationship studies on known and potential PtpA inhibitors to be pursued.

**Table 2.** Summary of antituberculotic compounds and drug candidates

Name	Class	Mechanism of action	Reference
**2-phospho-l-ascorbic acid**	Vitamin	Inhibitor of SapM via metal oxidation	[28]
**Aspirin**	NSAID*	Adjunctive host-directed therapy for pulmonary TB	[139, 195]
**AX20017**	Research compound	PknG inhibitor	[79]
**AZD4462**	Research compound	PknG inhibitor	[89, 92]
**AZD7762**	Research compound	PknG inhibitor	[90]
**Concanavalin A**	Research compound	Necrosis activator	[196]
**Cyclosporin A**	Immunosuppressant	Inhibitor of calcineurin	[44]
**FK506**	Immunosuppressive agent	Inhibitor of calcineurin	[44]
**Imatinib**	Cancer growth blocker	Inhibitor of Abelson tyrosine kinase	[28]
**Isoniazid**	Antitubercular Agent	Prodrug, cell-wall growth inhibitor	[124]
**L335-M34**	Research compound	PtpA inhibitor	[60]
**l-ascorbic acid**	Vitamin	Competitive inhibitor of SapM, redox destabilizer	[28]
**Loperamide**	Anti-motility (gut)	HDT, autophagy inducer	[197]
**Metformin**	Type 2-diabetic	HDT, oxidative stress inducer	[5]
**Nitazoxanide**	Anti-parasitic	Autophagy inhibitor	[198, 199]
**NU-6027**	Research compound	PknG inhibitor	[85]
**OA-NO_2_**	Research compound	PknG inhibitor	[95]
**Pretomanid/PA824**	Nitroimidazole	Nitric oxide stress inducer	[121, 122]
**R406**	Research compound	PknG inhibitor	[89]
**Rifampicin**	Antitubercular Agent	DNA replication inhibitor	[125]
**Saxifragifolin D**	Research compound	Accumulator of PI3K	[146]
**Sclerotiorin**	Natural product	Aldose reductase, lipoxygenase and PknG inhibitor	[84]
**Vitamin D3**	Vitamin	Adjunctive host-directed therapy for pulmonary TB	[139]

*NSAID: Non-steroidal anti-inflammatory.

Accumulating evidence suggests that PtkA has an important role in sensing and adapting to the dynamic environment during macrophage infection and should also be considered as a potential therapeutic target independent of PtpA. PtkA undergoes auto-phosphorylation, as well as serving as a substrate for several endogenous serine threonine protein kinases (STPKs) [50], which brings several challenges to chemically inactivating the protein. A recent review describes potential antimicrobial agents targeting STPK in Mtb [64]. The need for rapid development of novel antituberculosis drugs has seen researchers pursue drug repurposing screens, which enable the proposed compounds to reach clinical trials without repeating ISME protocols. One such study investigated the conformationally active, N-terminal intrinsically disordered domain (IDD) of PtkA as a screening target through high throughput virtual screening [65]. The study identified antiviral inosine pranobex and pain medication aesculin to have strong potential binding activity against PtkA. Follow-up studies regarding dose response under *in vitro* and *ex vivo* conditions are needed to confirm the compounds’ potential to inhibit Mtb growth.

Thioredoxin reductase (TrxB2), an important protein for oxidative stress defence, is also a substrate of PtkA [63]. Interestingly, the human homologue, TRXB2, has been speculated to play a major role in oxidative stress (OS) defence during Mtb infection, making compound development against TrxB2 difficult [66]. Although TrxB2 is essential for growth and survival of Mtb *in vitro*, and is needed for Mtb to establish and maintain infection in mice, partial inhibition of TrxB2 did not significantly increase the bacterium’s susceptibility to oxidative and nitrosative stress [67]. This suggests that TrxB2 has a more general buffering role in protecting against thiol-specific oxidizing stress and growth-essential processes such as DNA metabolism [67]. Remarkably, the Mtb Δ*ptkA* mutant presented increased secretion of TrxB2, making it more resistant to hydrogen peroxide compared to the parental strain [51]. This supports the notion that regulation by PtkA, due to its association not only with the PtpA but also with TrxB2, can serve at the front line for Mtb intracellular survival due to its association with redox defence.

### PknG

PknG is a member of the STPK family in Mtb [68]. Structurally, PknG is composed of a 4-cystein, metal ion-containing activating rubredoxin domain [69], a kinase domain and a tetratricopeptide repeat (TPR) [68, 70]. The *pknG* gene is part of a highly conserved glutamate metabolism operon, containing *glnX* and *glnH* [71, 72]. GlnH was shown to activate PknG via GlnX in response to the presence of amino acids Asp, Glu, and Asn in the extracellular space [73].

GarA was identified as a substrate for PknG [72], bestowing it with a postulated role in the regulation of the mycobacterial TCA cycle [48] and glutamine metabolism [71, 73]. As such, PknG confers survival advantage in hypoxic [74] or acidic [75] environments, suggesting that PknG is essential for Mtb pathogenesis *in vivo* due to its regulation of Mtb growth within infected cells and animals [71]. Additional PknG targets such as glutamine synthetase, FhaA and the 50S ribosomal protein L13 were identified in an *in vitro* interactome assay [76], and most recently, a mass spectrometry phosphoproteomics assay identified several potential additional substrates for PknG, reviewed in 2020 [77].

PknG is one of only two STPKs that are soluble, along with PknK [78] and the only reported STPK to be secreted from Mtb [71, 79]. As such, in addition to regulating metabolism in conditions encountered intracellularly, although still controversial, PknG was reported to inhibit phagosome-lysosome fusion [79] through several possible mechanisms. Compounds that specifically bind to and inhibit PknG, such as AX20017, restore phagosome-lysosome fusion and promote bacterial killing in macrophages [79]. Due to the STPKs similarity to eukaryotic kinases, PknG can possibly also act by targeting host STPK pathways. CypA, a human STPK, which can affect the NF-κB and ERK1/2 pathways was recently suggested as a PknG target in macrophages infected with *M. smegmatis* [80]. However, validation of this interaction in Mtb infections is still needed, and a link between CypA phosphorylation by PknG and blocking of phagosome-lysosome fusion has not been established. PknG was found to not interact with known phagosome-lysosome host factors (such as Rab5, PI3K3 and LAMP2 among others), and was suggested instead to interact with Rab7l1 [81], a host Golgi organization small GTPase associated with neuronal growth and Parkinson’s Disease [82]. However, until a well-supported host-mediated mechanism for inhibition of phagosome-lysosome fusion targeted by PknG can be found, the most plausible explanation is that disruptions to the mycobacterial TCA cycle result in the inability of Mtb to replicate intracellularly.

PknG has been a target for several drug-discovery efforts, including a 19 000 Nested Chemical Library screening campaign [83]. Using purified PknG, sclerotiorin [84] was identified as a nontoxic PknG inhibitor in BCG-infected macrophages, but not for growth in broth. This can be caused by the macrophage concentrating the compound in phagosomes, the compound being activated/metabolized only by the intracellular mycobacteria, the compound being activated by the host, or the target may be specific to the intracellular life stage of Mtb [84].

Cytotoxicity is a concern when developing drugs targeting eukaryotic kinases, and compounds targeting mycobacterial STPKs might also have non-specific binding sites in host kinases. Such an example can be seen in NU-6027, a host CDK1/2 inhibitor [85], recently reported to target PknD and PknG [86], but also inhibits host ATR and DNA-PK [87, 88]. The authors demonstrated that NU-6027 inhibits intracellular mycobacteria by promoting apoptosis in infected, but not in non-infected THP-1 cells, and was also effective in mice infected via aerosol, without observed toxic effects in the THP-1 model [86]. Conversely, AZD7762, another PknG inhibitor was toxic to THP-1 and J774.1 cells [89]. The same kinase library screen used to identify AZD4462 also discovered PknG inhibitors R406 and R406-free base, which showed no THP-1 cell cytotoxicity [89]. AZD7762 might be toxic via its inhibitory activity on the host checkpoint kinase 1 [90], while R406 is a spleen tyrosine kinase inhibitor [91]. Unlike sclerotiorin, both potentially host-targeting kinase inhibitors were also active on BCG growing in rich media [89]. This effect was not expected, as Δ*pknG* mutants show only limited growth reduction of Mtb in broth [71] and no growth reduction in BCG [92], which suggests these inhibitors may be promiscuous towards bacterial targets. Alternatively, the regulatory effect of PknG on mycobacterial metabolism during starvation conditions may explain these results [74, 93].

Fatty acid nitroalkenes are nitrated, unsaturated fatty acids, that, during metabolic and inflammatory reactions, modify protein thiols [94]. The 9- and 10-nitro-octadeca-9-cis-enoic acid (OA-NO_2_) was demonstrated to reversibly bind and irreversibly inhibit PknG, through the release of the ion from the rubredoxin domain [95]. It was not yet determined if OA-NO_2_ can, like other PknG inhibitors, restore phagolysosome fusion, perhaps due to drug-delivery issues. Still, OA-NO_2_ discovery highlights targeting the rubredoxin domain, rather than the kinase domain, might result in efficient PknG inhibitors that avoid the potential of cross-species kinase inhibition.

Overall, PknG is an intriguing drug target because of its essential role in Mtb basic metabolism and pathogenicity. PknG was successfully suppressed by inhibiting its kinase domain, though while risking secondary effects on both bacterial and host off-targets, or by modulating its regulatory rubredoxin domain. The multitude of compounds already demonstrated to reduce bacterial load by PknG inhibition highlight its viability as a drug target for novel compounds and for further optimization of existing compounds. For a recent review on Mtb STPK-targeting therapy see Khan and colleagues [96].

### Oxidative and nitrosative stress during phagocytosis

**Fig. 3.**
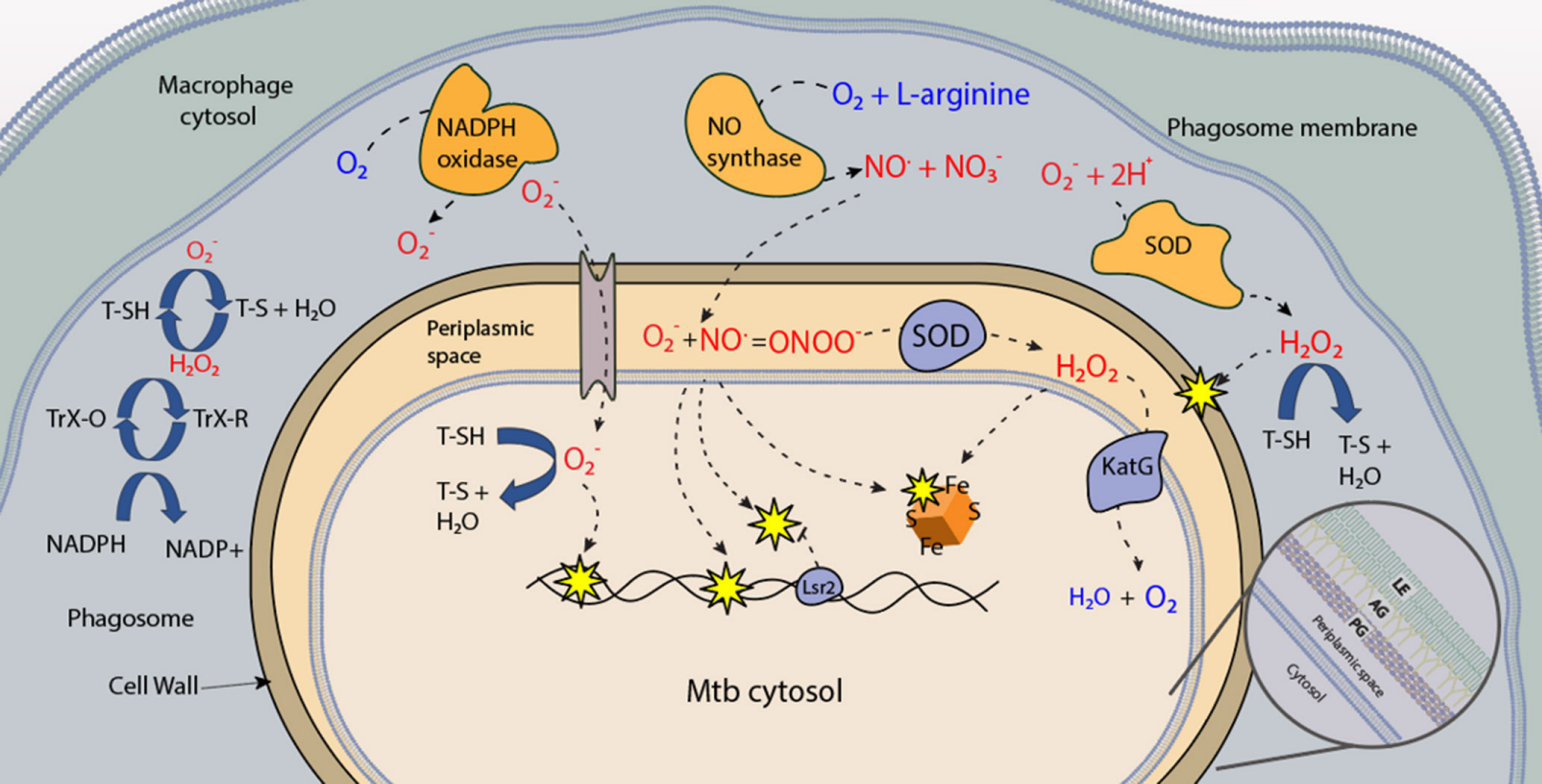
Stressors induced in the Mtb phagosome. During phagocytosis, the macrophage NADPH oxidase catalyses the production of peroxy-radicals, which are converted to hydrogen peroxide by superoxide dismutase (orange SOD). The peroxy-radical can enter Mtb through the anion channel, and damage bacterial DNA. Hydrogen peroxide can diffuse across the membrane and damage molecules such as iron-sulphur clusters, proteins or lipid in the membrane. Host inducible nitric oxide synthase (iNOS) will catalyse the release reactive nitrogen species. Mtb produces enzymes (blue) that can detoxify ROS and RNS. Mtb also produces low molecular thiols (T-SH), that detoxify RNS and ROS in the periplasm. Thioredoxin proteins that can reduce oxidized thiols, were shown to secreted. This figure depicts how secreted LMW thiols can detoxify ROS and RNS and are in turn recycled by TrX-Red (reduced), to maintain a less hostile extracellular environment. A detailed image of the mycobacterial cell envelope is included: PG=peptidoglycan, AG=arabinogalactan, LE=lipid envelope.

Oxidative and nitrosative induced stress (OS and NS) are characteristics of the toxic phagosomal environment during late-stage phagocytosis. An excessive release of reactive oxygen species (ROS) or reactive nitrogen species (RNS) results in the destruction of biological macromolecules such as DNA, proteins, lipids and others [97]. Although Mtb has developed mechanisms to block phagosome-lysosome fusion, it has also built the capacity to circumvent OS- and NS-induced damage (Fig. 3).

Interactions between Mtb PAMPs and the macrophage enables the activation of PRRs that induce NADP oxidase recruitment, leading to the generation of superoxide (O^-^_2_) peroxy radicals that are converted to perhydroxyl radicals (HO_2_^-^). HO_2_^-^ radicals have the ability to diffuse across the membrane and cause lipid peroxidation [98]. Superoxide dismutase (SOD) (Fig. 3) converts O^-^_2_ to hydrogen peroxide (H_2_O_2_) and dioxygen (O_2_) in the presence of water [99]. Concurrently, inducible nitric oxide synthase (iNOS) catalyses the conversion of l-arginine to nitric oxide and l-citruline. The produced nitric oxide (NO) can be subsequently converted to peroxynitrite [100]. These ROS and RNS generated during phagocytosis inhibit Mtb growth within macrophages, as recently reviewed in more detail [101]. However, Mtb can detoxify ROS/RNS, and consequently overcome the growth-inhibitory effects of OS/NS during phagocytosis. Mtb scavenges xenobiotics and free radicals using low molecular weight (LMW) thiols [102] and expresses antioxidative enzymes that serve as protectors during enzymatic detoxification.

Mtb synthesizes an array of unique LMW thiols, which each play an important role in orchestrating stress. The LMW thiol, glutathione (GSH), plays a versatile protective role in eukaryotes and has not been detected in mycobacteria [103, 104]. Instead, Mtb have been shown to synthesize alternative LMW thiols such as mycothiol (MSH) [105], gamma-glutamylcysteine (GGC) [106] and ergothioneine (ERG) [107], that interplay to protect against xenobiotics, ROS and RNS [108]. The MSH-deficient Δ*mshA* Mtb mutant displays a significant growth defect in human 1 macrophages after 3-to-6 days of infection [106], and a marginal growth defect in immunocompetent C57Bl/6 mice after 3 weeks of infection [109] but is indistinguishable from wild-type after 8 weeks of infection in the murine model [109]. Similarly, the ERG-deficient Δ*egtD* Mtb mutant showed a growth defect at both day 3 and 6 when infecting human THP-1 macrophages [106]. Δ*egtD* grew roughly a half-log less compared to wild-type in murine-derived macrophages at day 5 [107], and 5 weeks post-BALB/c mouse infection [110]. The only attenuated growth defect of the MSH-deficient mutants in animal models suggest compensation by other LMW thiols, supported by earlier studies showing elevated production of MSH in other thiol-deficient Mtb mutants [110, 111]. Further investigations demonstrated that Mtb mutants deficient in more than one LMW thiol had the most severe growth defect in both human and mouse macrophages [112]. These results confirm the interplay of LMW thiols to maximize the protection of Mtb and sustain its survival in macrophages, and highlight their potential as therapeutic targets.

In addition to LMW thiols, other detoxifying enzymes include the Cu-dependent SOD C and Fe-dependent SOD A superoxide dismutases [113]. Genetic interaction studies reveal that SOD A couples with the integral, yet unclassified membrane protein DoxX and a predicted thiol-oxidoreductase SseA to form the membrane-associated oxidoreductase complex (MRC) that contributes to LMW thiol homeostasis [114]. Another ROS detoxification enzyme that Mtb utilizes is the catalase peroxidase KatG, that is able to detoxify peroxidases but also activates the antibiotic INH (isonicotinic acid hydrazide/isoniazid) into a variety of active products including isonicotinic acyl NADH and biologically active NO [115, 116]. Furthermore, the akyl hydroperoxide reductase AhpC and the peroxiredoxin AhpE are able to detoxify hydrogen peroxide, organic peroxides, peroxynitrite and fatty acid hydroperoxides in order to maintain environmental homeostasis during infection [117]. In addition, Mtb can also detoxify RNS using the membrane-bound truncated haemoglobin N [118]. Mtb employs numerous enzymes involved in the detoxification of ROS and RNS, such as thiol peroxidases TrxB2 and TrxC, which act as disulphide reductases, reducing either oxidized LMW thiols and/or anti-oxidative enzymes such as alkyl peroxides [119, 120]. A more detailed enzymatic mechanism of these enzymes can be found in a recent review [101].

### Compounds that increase phagosomal-free radical stress in Mtb-infected macrophages

In view of the potential ability of Mtb to circumvent ROS/RNS during phagocytosis, a promising drug development approach is to investigate compounds that can increase host ROS/RNS during infection. Pretomanid (PA-824), a recently FDA-approved antimycobacterial drug, is a bicyclic nitroimidazole [121, 122], which is reduced by the deazaflavin (F_420_)-dependent nitroreductase (Ddn) into three metabolites, the major metabolite being des-nitroimidazole. These metabolites induce the release of RNS and NO, which consequently promote the anaerobic killing of Mtb [123]. Likewise, first-line TB drug INH, is a prodrug activated via oxidation by KatG [115]. The activated prodrug is then able to target mycolate biosynthesis in order to kill Mtb [124]. In addition to the inhibition of mycolate biosynthesis, the activation of INH is highly associated with the generation of ROS [116], which contributes to the anti-mycobacterial activity of INH. Co-treatment of Mtb with INH and an NO scavenger interferes with the anti-mycobacterial activity of INH [116]. In addition, RIF, the first-line TB drug that binds to the beta sub-unit of Mtb RNA polymerase and inhibits protein synthesis, is able to generate ROS *in vitro* [125] showing how drug-induced ROS generation already plays a key role in controlling Mtb infection in more classical antibiotics. Furthermore, vitamin C is a pro-oxidant, causing the release of ROS via the Harber-Weiss and Fenton reaction leading to a pleiotropic biological effect that consequently inhibits Mtb growth *in vitro* [37]. Though in the study vitamin C had no anti-mycobacterial activity in the murine model of infection alone, however when co-administered with INH and RIF, it decreased the bacterial burden in a synergistic manner [38].

Persisters are a small population of Mtb that remains after short-treatment regimens and are considered to be the main reservoir that dictates prolonged TB drug-therapy regimens [126, 127]. As Mtb is able to shut down its metabolism during certain conditions, antibiotics that are active against replicating bacteria or prodrugs that are required to be metabolized in order to be active are much less effective in these populations [128]. Vitamin C and pretomanid are suggested to target persistent Mtb by re-activating the metabolism of persistent populations [37, 123, 129]. Accordingly, treatment of Mtb with cysteine and other small thiols causes an increase in oxygen consumption and consequently an increase in ROS and antibiotic susceptibility [130]. This mechanism stimulates respiration in the metabolically inactive persisters, and consequently increase their susceptibility to compounds. Small thiols such as N-acetyl-cysteine have been shown to enhance the bactericidal effect of established first line drugs *in vitro* and *ex vivo* in murine macrophages [130]. However, in THP-1 cells, the anti-mycobacterial activity of N-acetyl-cysteine was shown to depend on its ability to decrease OS [131]. Both studies suggest that supplementing the TB drug regimen with small thiols has the potential to improve treatment success. While ROS and RNS damage intracellular Mtb, they also affect the host macrophage and modulate the host immune system, altering signalling and functions such as the recruitment of immune cells [132]. In light of this, the phytochemical thymoquinone (TQ) was shown to have anti-mycobacterial activity within macrophages due its ability to increase the level of NO and consequently affect the production of inflammatory cytokines [133]. In addition, GSH synthesized by the host is able to act as a nitric oxide carrier in the form of S-nitrosoglutathione [134]. This chemical property of GSH enables it to enhance the antimicrobial activity of immune cells during infection [135]. In order to develop appropriate ROS/RNS promoting drugs (pro-oxidants), it is imperative to better understand the host physiological factors that are influenced by and can interact with compounds that affect ROS/RNS during infection. For this reason, the molecular dynamics of the infected macrophage and the balance of host response to Mtb invasion should be considered. In the following section we will discuss host-directed therapies; a pertinent new avenue being explored the TB drug discovery efforts.

### Host-directed therapies

The swift rise in antibiotic resistance has seen the need for new and innovative ways to combat TB. Recently, the development of HDTs has come to the forefront of research against many intracellular pathogens [136]. HDT utilizes small molecules that target host responses instead of the pathogen itself, and has many benefits including proposed improved humoral memory and synergy with current antibiotics. Aside from clearing infections, HDTs are also able to modulate the immune response to limit inflammation and tissue damage associated with chronic infections. Indeed, although the lesser of two evils, cell-mediated damage during the prolonged and intensive chemotherapy required to treat tuberculosis is often just as damaging as the progressive disease itself. A recent paper discusses the impact that the extensive nature of TB treatment has on the microbiome of the host, and suggests HDTs may be a fruitful avenue to address this issue [137]. In the final section of this review, we will briefly discuss the rise and progress of novel HDT against Mtb.

Although the idea of HDT (reviewed recently in [138–140]), as Mtb targets is relatively new, the development of intracellular screening approaches [141, 142], and advancement in eukaryotic cell validation technologies, such as CRISPR and siRNA, are certain to boost HDT discoveries in TB therapy in coming years

HDTs are becoming an increasingly attractive avenue for adjunctive anti-tubercular treatment as resistance in the bacterium is unlikely to occur for a host-acting drugs [138, 143]. New technologies like high-content screening of intracellular Mtb show great promise in lessening the time needed to identify both host pathways that are being exploited by Mtb, as well as inhibitory compounds to target these mechanisms [141, 144, 145]. An additional benefit of a synergistic approach is decreased cytotoxicity of traditional chemotherapeutic agents and HDTs, if these compounds can be taken in lower concentrations adjunctively. Although the notion is relatively new, humans have been utilizing HDTs for centuries; saxifragifolin D for example, is a chemical derived from the plant *Androsace umbellate*, and has been used as a traditional Chinese medicine for cancer and bacterial infections for decades [146]. Recently, saxifragifolin D has been shown to increase active UVRAG-linked PI3K (a unique kinase also identified as VPS34 complex II) and prevent Mtb-induced phagosome maturation arrest in THP-1 macrophages by aiding in PI3P accumulation on the phagosomal membrane [146].

Chlorpromazine, an antipsychotic medication used to treat schizophrenia patients, is another candidate for HDT. It shows inhibition of Mtb survival in macrophages synergistically with first-line antibiotics, although in rather high concentrations [147]. A detailed review of the chlorpromazine class (Phenothiazines) compounds as mycobacterial treatment can be found by Kristiansen and colleagues [148]. Loperamide, another promising HDT candidate, has been shown to restrict intracellular growth of Mtb in lung macrophages via the activation of type I IFN and autophagic pathways, and was associated with the degradation of nuclear envelope protein p62 [149].

One of the pioneering cases on the use of an HDT to treat TB was with an FDA-approved leukaemia therapy and inhibitor of Abelson tyrosine kinase (ABL), imatinib (Gleevec). Imatinib was shown to have a synergistic effect in reducing both the number of granulomatous lesions and the bacterial load in infected organs when co-administered with the first-line drug RIF. Most interestingly, imatinib treatment was also effective against a RIF-resistant Mtb strains [150, 151]. The development of HDTs can also benefit from drug-repurposing of known signalling and regulation modulators to identify whether they have activity against and restrict Mtb growth. Investigations into existing FDA-approved drugs as HDTs are both economically and strategically wise. Metformin, for example, a drug widely used to treat type 2 diabetes, is an excellent example of a drug that has recently shown promise in reducing TB incidence. Metformin regulates oxidative phosphorylation, mTOR signalling and type I IFN response pathways. Following challenge with Mtb, metformin up-regulates genes involved in both phagocytosis and ROS production [152]. Importantly, the sum of these functions leads to the intrinsic enhancement of the T-cell response and better humoral memory [139]. For a recent review on metformin as an adjunctive TB therapy, see Naicker *et al.* [153]. Drugs first developed to control cancer growth have similarly been shown useful from screening for potential antimycobacterial agents [154]. A recent study by our group has revealed through the screening of kinase inhibitors on intracellular Mtb, that the CHK2 pathway is of importance to Mtb infection dynamics [155]. We reported the CHK2 inhibition by kinase inhibitor DDUG to be both strongly toxic to intracellular Mtb, and show low host-cell loss; a combination not seen in many campaigns against intracellular bacteria. Further investigation into the mechanism of inhibition needs to be performed, however siRNA knockdown confirmed the inhibitory phenotype in THP-1 macrophages [155].

### Host–pathogen cell fate in Mtb infection

Successful Mtb infection often leads to a myriad of proinflammatory events brought forth by the recognition of a buildup of, and a subsequent exposure to PAMPs by the immune system. Inflammatory cascades often lead to host-cell death, either by programmed and controlled cell death (apoptosis), or uncontrolled necrosis [156]. The inherent difference between these processes largely involves the amount of danger associated molecular patterns (DAMPs) and associated proinflammatory effector molecules that are exposed to the surrounding environment as the macrophage senesces. For a full review comparing DAMPs and PAMPs, see Tang *et al.* [157].

There is a particularly stark difference between apoptosis and necrosis as the former involves the entire apoptotic cell being engulfed by a secondary macrophage, almost entirely negating any extracellular DAMP escape and being for the most part, immunologically silent [158]. Immunogenic molecules can derive from Mtb itself (e.g. ESAT6) or the host (e.g. hydrolases and reactive species). Therefore, it is reasonable to state host systems favour apoptosis, while necrosis is most beneficial for Mtb, particularly in late infection [159]. This is explained as necrosis allows for greater Mtb proliferation, reinfection and dissemination over time, while apoptosis may be considered a second chance for the host to clear infection [160]. As the progression of apoptosis has been directly linked to successful mycobacterial clearance [161], Mtb has been shown to inhibit apoptosis at early stages of infection, favouring replication and dampening proinflammatory triggers, while also inducing necrosis at later stages of infection as a means of dissemination [159, 162].

Whether the cell-death pathway develops down a necrotic or apoptotic pathway depends on many complex and interacting factors of both the pathogen and host. It is pertinent to note that although there is a strain-dependent mechanism of apoptosis, the perception that avirulent Mtb drive apoptosis and virulent Mtb drive necrosis is outdated and simplistic. Although, there have been virulence-associated genes that affect the fate of cell death, including *secA2* and *nuoG*, as deletion of these genes shows host cell fate is driven towards apoptosis. However, multiple studies have contradicted the notion of virulence-associated cell fate by showing highly virulent Mtb strains often cause an apoptotic phenotype, and that apoptosis is more strongly associated with bacterial load than genetic determinants [160]. On the host–pathogen interaction front, evidence from our laboratory suggests that Mtb is able to block host apoptosis via PtpA-dependent dephosphorylation of host GSK3-α resulting in enhanced Mtb survival [46]. In contrast to the effects seen on GSK3α by PtpA that mediate down regulation of apoptosis, a recent study identified resveratrol, a Sirt1 activator, promotes Mtb clearance via apoptosis mediated by GSK3β [163]. The physiological benefit of Mtb’s avoidance of apoptosis via effector kinases is still under investigation; however, as apoptosis is a well-studied failsafe mechanism of protection from intracellular infection [162, 164], it is likely that the avoidance of cell death early in infection is integral for Mtb survival, replication and dissemination.

Macrophages that are unable to control intracellular Mtb replication have been shown to progress down an apoptotic path presumably to allow uptake by another, possibly more successful macrophage. This process not only provides a second chance for the host to control the infection, but also allows Mtb to disseminate around the tissue [160, 165]. Once an apoptotic cell is engulfed, the receiving macrophage moves along a chemotactic gradient to nascent granulomas where Mtb is both sequestered, and protected, such is the incongruity of the granuloma. Biochemically, the progression of each cell fate was reported to be mediated by prostaglandin E2 (PGE2) and lipoxin A4 (LXA4), in that PGE2 protects against necrosis and some strains of Mtb inhibit PGE2 while inducing LX4A, leading to necrosis [166]. It is also true that via the inhibition of apoptosis over time, the only remaining outcome for the macrophage is necrosis and as such, whether Mtb directly drives necrosis in macrophages is open to some debate. It has been shown that only actively replicating mycobacteria are able to drive the cell-death pathway towards either necrosis or apoptosis, and that lung epithelial cells undergo a strongly necrotic cell death under Mtb infection [167], meaning actively secreted effectors may play an important role in the management of apoptosis.

Autophagy is another well-documented event that can either help the host cell eradicate Mtb or conversely, aid Mtb to replicate in a membrane-encapsulated niche via the autophagosome [168–170]. Macroautophagy (referred to as autophagy in the context of this manuscript) is a process characterized by the formation of a double-membrane vesicle, termed the autophagosome. The autophagosome functions as a cellular recycling mechanism by engulfing components of the cytoplasm, including phagosomes, with the terminal outcome being delivery to lysosomes for degradation. Autophagy is not only imperative for the maintenance and recycling of cellular constituents, but has recently been shown to be a crucial mechanism of the control of many pathogenic micro-organisms, including mycobacteria, *S. aureus, Shigella* spp*., Salmonella* spp*.* and *Rickettsia* spp*.* This process was termed xenophagy and was first suggested as a potential means of mycobacterial control in 1997 [171], also reviewed by Hu *et al.* [172]. Autophagic manipulation was further linked to Mtb when the induction of autophagy was associated with pro-inflammatory cytokines similar to those produced during Mtb infection. Rationale depicts that although there were pro-autophagic drivers in play, intracellular Mtb remained predominantly in single membrane-bound vesicles, suggesting the presence of anti-autophagic strategies used by Mtb. One of the main components of autophagy induction is IFN-γ, which has been shown to be modulated during Mtb infection [173]. Autophagy can be stimulated by a variety of signals, both environmental and chemical. The most well-researched mechanisms of autophagy induction include nutrient starvation, oxidative stress, Mtb infection and chemical stimulation (listed in Table 3). An important consideration for the development of autophagy-inducing TB therapies is the immunosuppressive response caused by the direct inhibition of mTOR, the main master regulator of autophagy. As most autophagy targeting compounds modulate the effects of mTOR, the need to find mTOR-independent autophagy-mediating inhibitors have been explored. The drug carbamazepine has been shown to induce autophagy via an mTOR independent route [174]. Pasakbumin A (Pas A), a compound derived from the plant *Eurycoma longifolia*, has been reported to inhibit intracellular Mtb growth by inducing autophagy via the ERK1/2-mediated signalling pathway [175]. The authors describe synergy with RIF, however the data show only a modest change in dose response with the addition of Pas A, coupled with a 50 % intracellular Mtb reduction at 10 µM Pas A alone, suggests this compound may be a more fruitful candidate after structure activity relationship dynamics have been explored.

**Table 3.** Potential TB therapeutic compounds targeting host cell recycling and death responses

Drug name	Drug class	Response affected (pos/neg response)	Reference
**Levofloxacin**	Fluoroquinolone antibiotic	Apoptosis (+)	[200]
**Rapamycin**	Macrolide antibiotic	Apoptosis (+)	[201]
**Carbamazepine**	Anti-convulsant	Autophagy (+)	[174]
**Isoniazid**	Antitubercular antibiotic	Autophagy (+)	[181]
**Loperamide**	Anti-diarrheal	Autophagy (+)	[197]
**Nitazoxanide**	Anti-parasitic	Autophagy (+)	[198, 199]
**Pyrazinamide**	Antitubercular antibiotic	Autophagy (+)	[181]
**Streptomycin**	Aminoglycoside antibiotic	Necrosis (-)	[167]

Autophagy, like apoptosis and necrosis, also largely determines the type of immune response and inflammatory signals released by both the infected host cell and surrounding cells. Here, rather than presenting a topic that is well-reviewed [176–180], we wish to highlight the benefit of using targeted compounds to specifically activate each of the pathways. Compounds including repurposed FDA approved drugs were shown to reduce Mtb intracellular load that affect each of the cell-fate pathways and their targets are summarized in Table 3. Of note is the inclusion of current first- and second-line therapies, which have been shown to induce, and have their activity dependent on the autophagic pathway [181]. We review here the outstanding ability of Mtb to not only survive inside the phagosome by producing effectors that balance unfavourable conditions, but also act on the host to delay or inhibit the development of the phagosome maturation pathway, and most recently described, the ability to drive the overall cell fate of the macrophage to favour survival. This capability to ensure persistence by adapting to, and changing the host environment on three levels (environmental protection strategies, host response defence and interrupting host cell-fate pathways) highlights well the dynamic nature of host–pathogen interactions from a bacterium that has lived with humans for millennia.

## Conclusions

Understandings of Mtb pathogenesis over the last decade have seen significant developments, particularly in the scope of molecular biology of host–pathogen interactions. With Mtb initiating lifelong infection inside resident immune cells, the need to consider its unique intracellular biochemistry and advance on classical antibiotic research into novel screening and drug development is imperative for the elimination of TB this century. As discussed, one of the cornerstones of Mtb pathogenesis is its ability to sustain life inside the early phagosome of the macrophage, achieved in the multistep mechanism; phagosome maturation arrest. Researchers have been able to develop several novel inhibitors for the phagosome maturation arrest pathway mediated by Mtb. This includes common vitamin C as a pro-oxidant and an inhibitor of SapM to research compounds specifically screened to affect PtpA and other secreted effectors. As PtpA is the main driver of phagosome maturation arrest and works not only by dually inhibiting the acidification of the phagosome as well as preventing lysosome docking and subsequent merging, but also affects host cell fate and metabolism, defined by effected signalling activity it is plausible that further screening for inhibitors is a worthwhile endeavour. Inhibiting signalling substrates of PtpA can help identify novel HDT host-acting properties. Indeed molecular inhibition of intracellular signalling is being increasingly pursued as a means of pathogen clearance across many genera [182], leading to development of drugs which modulate host responses to avoid host cell death mediated by Mtb PtpA and the ESX secretion system [46, 183, 184].

During phagocytosis, ROS and RNS released by macrophages play a versatile role. They not only damage the invading pathogen, but also modulate, either directly or indirectly, cytokine production thereby enhancing the antibacterial activity of the immune cells. Thus, LMW thiols that are able to scavenge ROS and RNS, and anti-oxidative enzymes that participate in the process also become a promising target for TB drug development. Most importantly is that targeting LMW thiols may give rise to drugs active against the metabolically dormant persisters, which are typically the main cause for prolonged and oftentimes damaging TB regimens.

Carrying an active hit compound from bench-top to clinic is time consuming and often promising drugs leave the pipeline due to economic reasons independent of treatment success. To counter the bottleneck associated with novel compound development, researchers and pharmaceutical agencies are looking into repurposing pre-existing and pre-approved drugs that show activity against Mtb, or alternatively reduce the cell-mediated tissue damage associated with chronic TB. For example, recent FDA-approved, re-classified drugs, linezolid and metformin have the potential to save tens of millions of lives as they are active against drug-resistant TB. The re-classification of linezolid (originally permitted for use against Gram-positive bacteria), and metformin (a type II diabetes therapy) has proven wise, as both have been shown to act on drug-resistant pulmonary tuberculosis [185, 186]. Metformin, aspirin and vitamin D3 are all suggested adjunctive therapies during the treatment of tuberculosis [139]. More research is needed to elucidate the complex interactions between pathogen and host, particularly due to the low numbers of anti-Mtb compounds being approved by the FDA in previous decades. Focusing on changing the perspective by which pharmaceuticals are discovered is an important driver of progress in the new century and has already demonstrated promising results in the fields of cancer and infectious disease.

Overall, we wish to promote the consideration of adjunctive therapy for the treatment of Mtb. By incorporating effector-specific modulation of pathogen, coupled with targeting host-specific processes alongside broad-acting, classical antibiotics, it is possible that TB treatment could be significantly reduced in treatment time and associated cellular damage. Taking into consideration the intracellular nature of Mtb during drug development and screening is invaluable for researchers worldwide, this includes the elucidation of host kinase interactions as promising avenues for novel therapy. With the rise of MDR-TB, investigations into known safe drugs is also a valuable strategy for the desperately needed end of TB worldwide.
